# Process Chain for Ultra-Precision and High-Efficiency Manufacturing of Large-Aperture Silicon Carbide Aspheric Mirrors

**DOI:** 10.3390/mi14040737

**Published:** 2023-03-27

**Authors:** Bo Zhong, Wei Wu, Jian Wang, Lian Zhou, Jing Hou, Baojian Ji, Wenhui Deng, Qiancai Wei, Chunjin Wang, Qiao Xu

**Affiliations:** 1 Laser Fusion Research Center, China Academy of Engineering Physics, Mianyang 621900, China; zhongbo_foerc@163.com (B.Z.);; 2 State Key Laboratory of Ultra-Precision Machining Technology, Department of Industrial and Systems Engineering, The Hong Kong Polytechnic University, Hung Hom, Hong Kong 999077, China

**Keywords:** silicon carbide, aspheric mirror, ultra-precision shaping, deterministic polishing, precision testing

## Abstract

A large-aperture silicon carbide (SiC) aspheric mirror has the advantages of being light weight and having a high specific stiffness, which is the key component of a space optical system. However, SiC has the characteristics of high hardness and multi-component, which makes it difficult to realize efficient, high-precision, and low-defect processing. To solve this problem, a novel process chain combining ultra-precision shaping based on parallel grinding, rapid polishing with central fluid supply, and magnetorheological finishing (MRF) is proposed in this paper. The key technologies include the passivation and life prediction of the wheel in SiC ultra-precision grinding (UPG), the generation and suppression mechanism of pit defects on the SiC surface, deterministic and ultra-smooth polishing by MRF, and compensation interference detection of the high-order aspheric surface by a computer-generated hologram (CGH). The verification experiment was conducted on a Ø460 mm SiC aspheric mirror, whose initial surface shape error was 4.15 μm in peak-to-valley (PV) and a root-mean-square roughness (Rq) of 44.56 nm. After conducting the proposed process chain, a surface error of RMS 7.42 nm and a Rq of 0.33 nm were successfully obtained. Moreover, the whole processing cycle is only about 216 h, which sheds light on the mass production of large-aperture silicon carbide aspheric mirrors.

## 1. Introduction

With the continuous development of space science and technology, the performance of space optical systems has gradually improved, which has put forward increasingly strict requirements on the working band, imaging resolution, thermal stability, and system weight of optical systems, thereby promoting the development of optical systems in the large aperture, reflection, and lightweight directions [1,2]. Silicon carbide (SiC) material has the characteristics of low density, high modulus, high specific stiffness, high dimensional stability, and good thermal properties and has become the preferred material for the new generation of space reflectors [3]. Currently, large aperture silicon carbide (SiC) aspheric mirrors have been used in space optical remote sensing detection systems, high-resolution ground observation systems, space telescopes, and other space optical systems such as the SPICA infrared astronomical telescope [4], the ASTRO-F astronomical telescope [5], and the JWST space telescope [6].

However, due to its high hardness and complex composition compared to traditional optical glass, SiC material is difficult to process with high efficiency, precision, and an ultra-smooth surface [7]. In particular, the machining of large, lightweight, and aspherical SiC mirrors is still a major challenge in the field of optical manufacturing, which involves high difficulty and long cycle times. In recent decades, extensive research has been conducted on SiC mirror optical processing worldwide, accumulating a wealth of experience and achieving some results. An American SSG company developed the ALI optical subsystem for NASA, with both its primary mirror and tertiary mirror being coated silicon RB-SiC reflecting mirrors. The surface form accuracy of the main mirror was as high as 0.035 λ (λ = 632.8 nm), and the surface roughness RMS was 1–1.5 nm [8]. SiC reflective mirrors prepared by the reaction sintering method developed by SSG [9] in the United States were applied to space telescopes such as the Miniature Infrared Camera and Spectrometer (MICAS) and the Advanced Land Imager (ALI), both of which are important parts of NASA’s NMPDS-1 (New Millennium Program Deep Space-1) project. The PV value of the SiC reflective mirror was 0.7 λ and the RMS was 0.13 λ (λ = 632.8 nm). Litton Itek and HDOS (Hughes Danbury Optical Systems) in the United States have used reaction-sintered SiC material to produce lightweight mirrors. Breidenthal et al. from Litton Itek [10] used reaction-sintering to produce a SiC mirror with dimensions of 1125 mm × 825 mm. The surface shape accuracy after polishing was λ/20, and the surface roughness was 3 nm. ASTRIUM used ion beam figuring (IBF) technology to process SiC mirrors, and the upper-level reflecting mirror of the SOFIA telescope is an aspherical SiC mirror with an open back structure, with a diameter of 352 mm and a thickness of 40 mm. The surface shape accuracy after IBF is 39 nm RMS [11]. The S.I. Vavilov State Optical Institute in Russia developed a SiC-Si biphasic material called Sicar using the RB method. The institute used Sicar to manufacture honeycomb-structured mirrors with a diameter of 170 mm and closed-back mirrors with dimensions of 308 mm × 210 mm. The RMS surface accuracy achieved for these mirrors is between ±0.02 λ and ±0.04 λ [12]. Hang [13] employed a coupled process of CCOS and MRF techniques to modify the surface of an off-axis aspherical SiC mirror with a diameter of 540 mm. After 33 h of polishing, the modified layer improved the surface accuracy while also controlling the mid-to-high-frequency errors, resulting in a final PV value of 0.486 λ and an RMS of 0.018 λ. Wang et al. [14] conducted milling, grinding, rough polishing, and precision polishing on an octagonal off-axis silicon carbide aspheric surface with a dimension of 600 mm × 270 mm, whose RMS value is better than 1/50 λ. Zhang et al. [15] used precision milling technology to process a 900 mm × 660 mm off-axis SiC aspheric mirror, with a surface shape accuracy PV of 18.8 µm and a RMS 3.5 µm after processing. Zhang et al. [16] combined computer numerical control polishing, flexible chemical mechanical polishing, and IBF to process a 600 mm × 200 mm modified carbonized silicon flat mirror for a space camera. The RMS surface accuracy achieved was 0.014 λ (λ = 632.8 nm), and the surface roughness was 0.71 nm. Zhang et al. [17] employed computer-controlled optical surface processing (CCOS) technology to fabricate a 650 mm × 200 mm SiC mirror, achieving a higher polishing efficiency with CeO_2_ polishing agent and the best optical surface using a SiO_2_ polishing agent. The surface accuracy of the modified aspherical SiC mirror was 0.016 λ (RMS), and the surface roughness was 0.85 nm (RMS). Song et al. [18] improved the processing stability by optimizing the grinding tool, and after four rounds of about 40 h of processing (634 mm × 560 mm), SiC was off-axis spherical with a machining error of 239 λ that was improved to 115.32 λ. Zhang et al. [19] recently reported the strategies for the fabrication of the world’s largest SiC aspheric mirror, with a size of Ø4.03 m. Extremely high surface accuracy of this mirror was obtained after the whole fabrication process, whose final surface figure error and roughness were 15.2 nm RMS and 0.8 nm RMS, respectively. While the production efficiency is still not high enough, which can take several months.

As mentioned above, processing methods based on advanced optical manufacturing technologies such as MRF and IBF have greatly improved the manufacturing accuracy of large-aperture silicon carbide optical components, and the ultimate manufacturing accuracy can reach the nanometer level. However, research on ultra-precision shaping and high-efficiency polishing of large-aperture silicon carbide aspherical mirrors is still limited, and the overall manufacturing efficiency is still relatively low. Currently, the entire process of manufacturing large-aperture silicon carbide aspherical mirrors is still imperfect, for example, because of the low efficiency and precision of shaping [15,18], the low efficiency and insufficient stability of polishing [20,21], the tendency for the silicon carbide substrate to form concave pit defects [22,23], and the tendency for comet-like defects to form on the modified silicon surface [24], resulting in a low overall manufacturing efficiency (the manufacturing cycle of large-aperture silicon carbide aspherical mirrors can be several months or more) and an inability to meet the increasing demand for rapidly deploying space optical systems. This paper proposes an efficient manufacturing process chain combining ultra-precision grinding, rapid polishing, central fluid supply, and magnetorheological finishing (MRF), aiming at the high-precision and batch manufacturing requirements of large-aperture silicon carbide aspheric mirrors in space optical systems, and it has been experimentally proven to be effective in improving the manufacturing accuracy and efficiency of large-aperture silicon carbide aspheric mirrors.

## 2. Ultra-Precision Manufacturing Technology for Large Aperture Silicon Carbide Aspheric Mirrors

To meet the demand for batch manufacturing of aspherical surfaces, the research team proposed a strong laser-based ultra-precision manufacturing process with “ultra-precision and determinism” as its core [25]. After years of research and exploration, a relatively mature aspherical surface processing process has been developed, including aspherical surface grinding, and shaping, rapid polishing, and precision polishing stages. In the ultra-precision grinding and shaping stage, micro-level surface accuracy is directly obtained through parallel grinding technology while controlling defects. In the rapid polishing stage, high-efficiency polishing technology is used to remove grinding ripples and defects, quickly correct the surface shape to meet the requirements of precision polishing, and suppress processing defects. In the precision polishing stage, high-deterministic technology is used to correct the surface accuracy to nanometer-level accuracy, with surface roughness reaching sub-nanometer accuracy. The characteristics of this process route are based on the ideas of accuracy decomposition and efficiency matching. By combining ultra-precision shaping technology with various deterministic sub-aperture polishing technologies, the advantages of unit technology are fully utilized to achieve quantifiable control over the entire process in terms of accuracy, efficiency, and defects.

**Figure 1:**
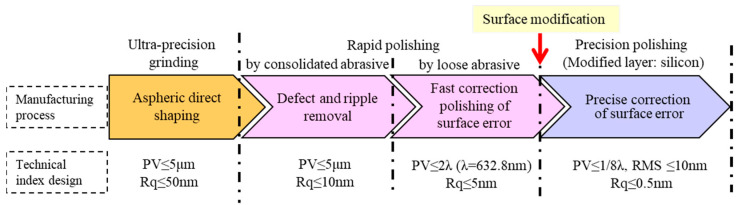
Design of an ultra-precision manufacturing process for large-aperture silicon carbide aspheric mirrors.

As a typical aspherical component, large-aperture silicon carbide aspheric mirrors are also applicable to the main process route mentioned above. However, the technical scheme needs to be optimized according to the characteristics of silicon carbide aspheric mirrors, such as hard materials and lightweight structures. In order to obtain the designed optical function, the asphericity of silicon carbide mirrors is generally large, and a structurally unstable layer is formed on the surface of the mirror blank during sintering, resulting in a large amount of material removal. Therefore, the most efficient way is to use ultra-precision grinding to remove the surface layer of the mirror blank and directly obtain a micrometer-level aspherical shape, which will greatly reduce the processing margin in the subsequent polishing stage. Aspherical grinding and shaping are necessary processing methods for the efficient manufacturing of silicon carbide mirrors, with the main focus on the blunting problem of the grinding wheel for hard materials. Due to the high hardness of silicon carbide materials, traditional polishing methods have low removal efficiency. Therefore, a small tool based on CNC polishing technology and bonded abrasives is proposed to quickly remove grinding ripples and defects. In addition, RB-SiC is a multiphase structure, and the traditional high-speed and high-pressure processing method can easily cause “pitting” defects due to thermal effects at the processing interface. Therefore, a small tool CNC polishing technology based on center-supplying liquid is proposed to suppress the thermal effects at the processing interface and thus suppress the defects on the silicon carbide substrate. In the final precision polishing stage, the modified silicon layer is the target of processing. The silicon surface layer is prone to comet-like defects, and a magnetorheological polishing technology with low stress and high determinism is used for precise shaping. The ultra-precision manufacturing process for large-aperture silicon carbide aspheric mirrors is shown in Figure 1.

## 3. Key Technologies for Ultra-Precision Manufacturing of Large-Aperture Aspheric Silicon Carbide Mirrors

### 3.1. Ultra-Precision Grinding Wheel Blunting and Life Prediction

The Mohs hardness of silicon carbide material exceeds 9. During the grinding process of large-aperture silicon carbide mirrors, diamond grinding wheels are prone to wear and dullness [26], resulting in a sharp increase in grinding force, affecting the shaping accuracy of the component, and reducing the surface quality. To achieve ultra-precision shaping and processing of large-aperture aspherical silicon carbide components, it is necessary to clarify the wear failure mechanism of the diamond grinding wheel during the grinding process, establish the evolution law of grinding wheel wear, and quantitatively predict the service life of the grinding wheel.

(a) Wear and failure mechanisms of a silicon carbide grinding wheel.

**Figure 2:**
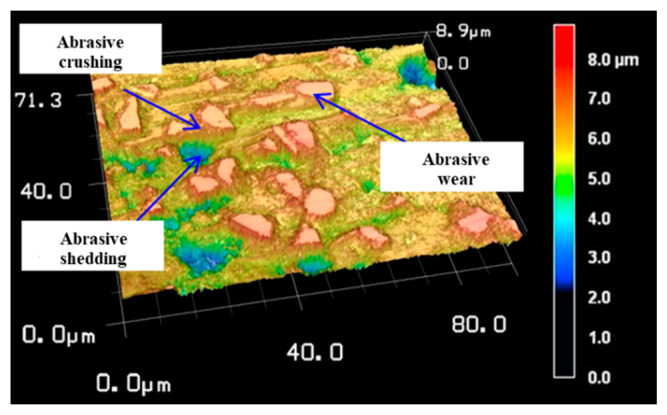
Microscopic wear of a silicon carbide grinding wheel.

**Figure 3:**
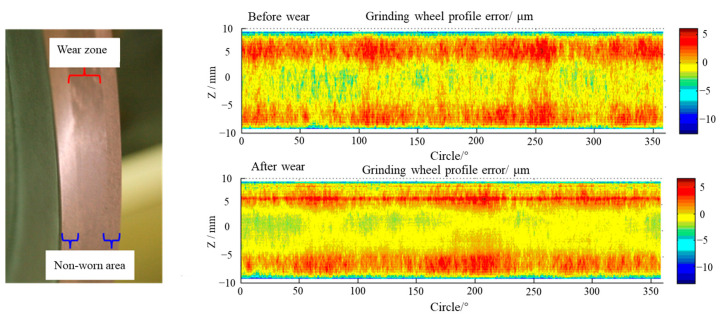
Macroscopic wear morphology of a silicon carbide grinding wheel.

Through experiments using arc-profiled diamond abrasive wheels for grinding silicon carbide materials, it was found that the silicon carbide material was removed through brittle fracture under the action of diamond abrasives. The surface morphology of the worn diamond abrasive wheel is shown in Figure 2. It can be seen that during the interaction between the diamond abrasives and the silicon carbide material, the micro-wear form of the wheel mainly exhibits abrasive wear. With the intensification of wear, the individual abrasive particles experience an increased reactive force from the component, gradually leading to the phenomenon of abrasive particle fracture and detachment. During the aspherical machining process using an arc-profiled grinding wheel along parallel machining tracks, since the component contact point with the grinding wheel surface is tangent to the point of processing, the entire arc-profiled grinding wheel section is involved in the grinding process and exhibits uniform wear, as shown in Figure 3, which depicts the macroscopic wear morphology of the silicon carbide grinding wheel. It can be seen that only slight changes in geometric dimensions occur in the wear area. Through experiments, it was found that the wear failure mechanism of the silicon carbide grinding wheel is mainly due to the micro-wear of the diamond abrasive grains. As the exposed edges of the abrasive particles become dulled, the grinding force increases, affecting both the surface roughness and the depth of crack defects on the component surface. It can also lead to significant elastic deformation of the lightweight silicon carbide component and affect the final processing accuracy.

(b) Wear pattern and life prediction of silicon carbide grinding wheels.

**Figure 4:**
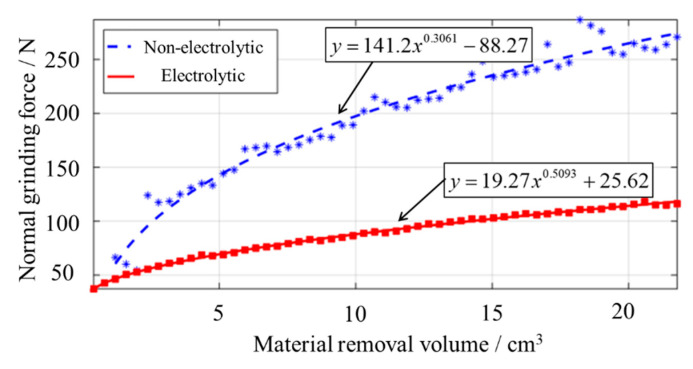
Evolution of grinding force during grinding of silicon carbide material.

Grinding force is the key factor that affects the shaping accuracy and quality of SiC aspheric mirrors with lightweight structures. The grinding force is related to the abrasive wear characteristics of the grinding wheel and the working time (i.e., the degree of grinding wheel wear). Working time under fixed process conditions can also be equivalent to cumulative material removal. Therefore, there are two core process requirements for the high-precision forming and processing of large-aperture SiC mirrors. One is to improve the wear resistance of the grinding wheel, and the other is to clarify the cumulative removal amount within the wheel life and complete the machining within the service life of the wheel. In response to the above requirements, a diamond grinding wheel wear experiment was conducted in which common processing parameters were selected, namely the grinding wheel linear speed of 30 m/s, the feed speed of 5000 mm/min, and the grinding depth of 10 μm per time. The parameters of the diamond grinding wheel are 400 mm in diameter, 20 mm in width, and 8–12 μm in grain size. The electrolytic parameters of the grinding wheel are DC 24 V, current 2 A, and electrolytic time 30 min. Under the same process parameters, metal-bonded diamond grinding wheels with and without electrolytic sharpening were used to process silicon carbide components, and the grinding forces at different cumulative removal volumes of silicon carbide materials were measured simultaneously, as shown in Figure 4. From the experimental results, it can be seen that as the cumulative volume of material removal increases, the grinding force gradually increases, and the grinding wheel wear gradually intensifies. For the diamond grinding wheel that has undergone electrolytic sharpening, the microprotrusions of the diamond abrasive particles are effective, and the abrasive particle sharpness is high. The grinding force at the same cumulative volume of material removal is significantly smaller than that of the grinding wheel without electrolytic sharpening, and the growth rate is also significantly lower than that of the electrolytic wheel. After the arc-shaped diamond grinding wheel is dressed, further electrolytic sharpening is performed to selectively remove the metal binder on the surface of the grinding wheel and expose the diamond abrasive particles. This can greatly improve the processing performance of the grinding wheel and reduce the grinding force. Using the grinding force fitting model obtained from the experiment and the cumulative volume of material removal as the quantified evaluation index of grinding wheel wear, the grinding force of the same type of diamond grinding wheel at different machining stages under the same grinding parameters can be accurately predicted.

This text describes the experiment and processing of aspherical silicon carbide mirrors for lightweight structures, combined with non-contact displacement sensors to measure the force deformation at different positions on the surface of the component. The experiment found that when a normal force of 70 N is applied at the central position of the lightweight hole of the silicon carbide mirror, the deformation at that position has reached about 1μm, which has already affected the processing accuracy of the component. Therefore, to ensure the processing accuracy of the component, it is necessary to ensure that the normal grinding force during the entire grinding process does not exceed 70 N. Based on the evolution law of the silicon carbide material grinding wheel wear in Figure 4, on the one hand, an electrolytically trimmed diamond grinding wheel is selected for grinding, and on the other hand, the cumulative volume of material removed is controlled not to exceed 5.13 cm^3^ as the criteria for grinding wheel life. The above research shows that electrolytic grinding wheels have stronger wear resistance, which can inhibit the rapid increase in grinding force, thereby ensuring the micron-level shaping accuracy of large-aperture SiC aspherical surfaces during long-term processing (within the lifetime of the electrolytic grinding wheel).

### 3.2. Low-Defect Rapid Polishing

(a) Efficiency of center-feed rapid polishing

**Figure 5:**
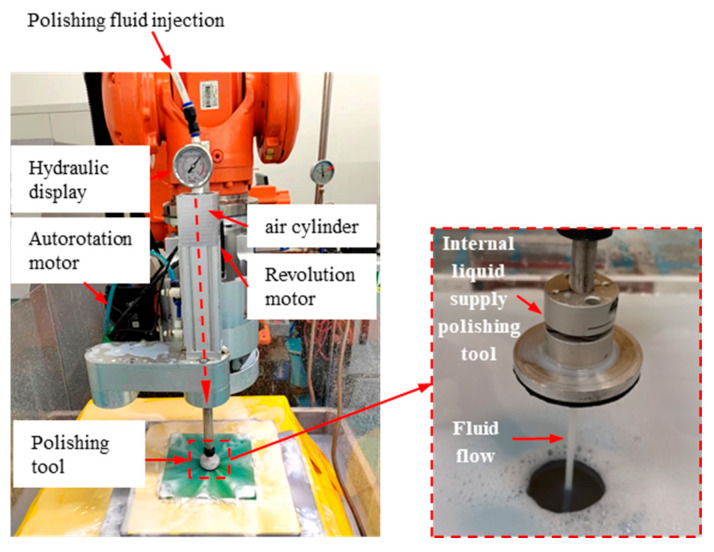
Center liquid supply small tool polishing device.

In order to improve the efficiency of aspherical polishing, a more stable polishing mechanism was designed in the early stages to significantly increase the rotational speed and polishing pressure of the polishing tool (the rotational speed was increased from 200 rpm to 3000 rpm, and the pressure was increased from 20 N to 120 N) to improve efficiency. However, experiments found that increasing the process parameters did not result in efficiency improvements consistent with the theory. It was speculated that in the high-speed and high-pressure machining process, it was difficult for the polishing liquid to enter the bottom of the polishing tool, resulting in fewer polishing particles participating in the grinding and polishing, which affected the polishing efficiency. In addition, because it was difficult for the polishing liquid to enter the central area of the bottom of the tool, the heat generated by the friction between the tool and the component could not be taken away, increasing the temperature in the central area of the bottom of the tool, accelerating tool wear, and further affecting the stability of material removal. To address these issues, a center-feed CNC polishing device was introduced [27], as shown in Figure 5. The device includes a public rotation motor, a hollow cylinder, a hydraulic display gauge, and an internally fed polishing tool. The upper end of the hollow rotating shaft in the cylinder is connected to the polishing liquid injection pipe through a rotating slip ring. When the hollow rotating shaft rotates at high speed, the polishing liquid injection pipe does not rotate. The lower end of the hollow rotating shaft is connected to the hollow polishing disc. The public rotation motor drives the polishing disc to move in a planetary motion. The polishing liquid enters the polishing contact area through the injection pipe, the hollow shaft, and the hollow polishing disc, which is conducive to updating the polishing liquid and taking away the machining heat.

**Figure 6:**
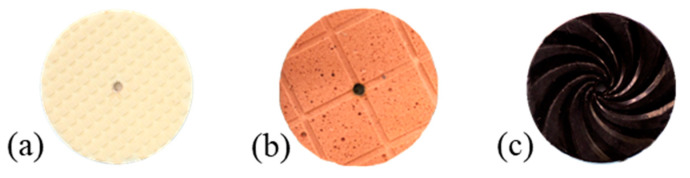
Three types of polishing tools. (**a**) Consolidated abrasive; (**b**) polyurethane; (**c**) asphalt.

**Figure 7:**
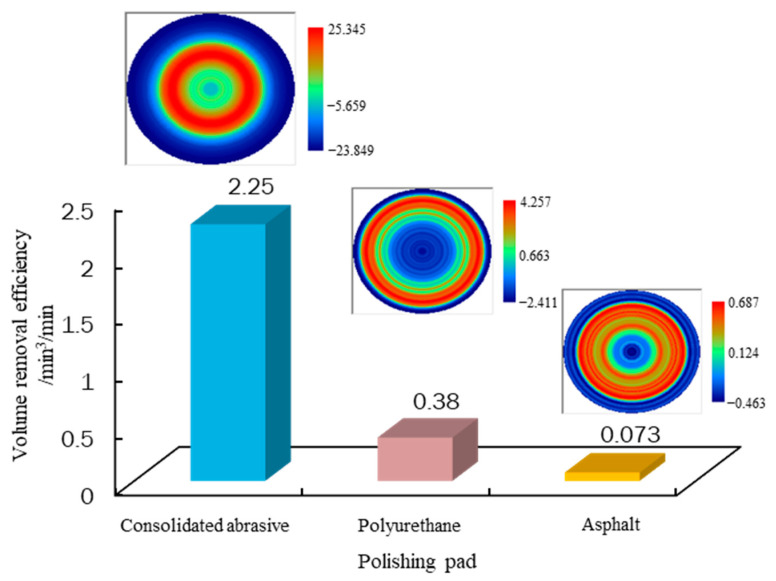
Comparison of the removal efficiency of different polishing tools.

To obtain different removal characteristics, the polishing disc mold layer uses sintered abrasive, polyurethane, and asphalt, as shown in Figure 6. The above three polishing molds with central supply polishing tools were used for spot experiments on silicon carbide test substrates. Other experimental parameters remained the same, with a tool diameter of Ø50 mm, a diamond abrasive particle size of 3 μm, a solution concentration of 1%, a polishing pressure of 45 N, a tool speed of 1000 rpm, and a processing time of 10 s. After the experiment, the polished spot was collected using a flat interferometer, and the volume removal efficiency was calculated as shown in Figure 7.

According to Figure 7, the volume removal rates of the three polishing models, namely sintered abrasive, polyurethane, and asphalt, are 2.25 mm^3^/min, 0.38 mm^3^/min, and 0.073 mm^3^/min, respectively. It can be seen that the sintered abrasive has the highest removal efficiency in processing SiC materials, which is six times that of polyurethane and thirty times that of asphalt. To meet the process requirements of “efficient removal of grinding defects and ripples”, “rapid shape correction”, and “quick improvement of smoothness” in the rapid polishing stage, a consolidated abrasive tool is used to quickly remove ripples and defects, a polyurethane tool is used for rapid shape correction and the removal of primary knife marks; and an asphalt tool is used for further improving the surface roughness. Based on the processing characteristics of three-layer materials, a combination of polishing tools with different layer materials can fully meet the requirements of the rapid polishing stage, while the consolidated abrasive tool greatly improves processing efficiency.

(b) Generation and suppression mechanism of “pit” defects in SiC

**Figure 8:**
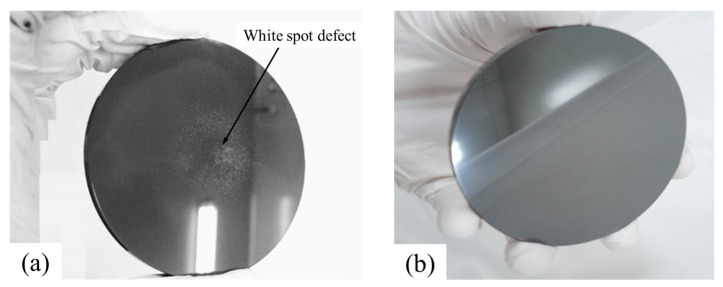
Comparison chart of surface quality with different liquid supply methods. (**a**) External liquid supply; (**b**) internal liquid supply.

**Figure 9:**
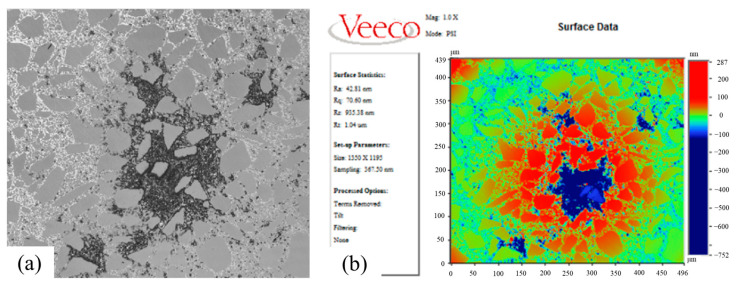
Microscopic imaging of the surface of externally supplied machining components. (**a**) Micrograph; (**b**) roughness.

**Figure 10:**
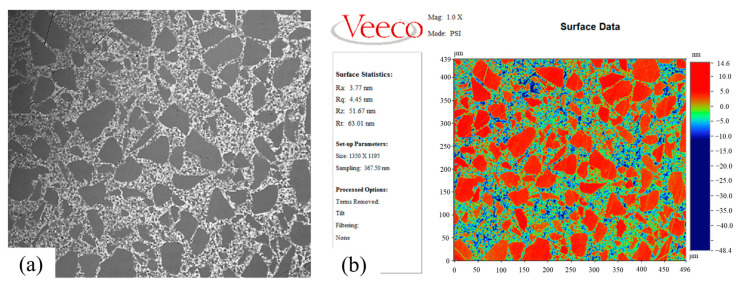
Microscopic imaging of the surface of internally supplied machining components. (**a**) Micrograph; (**b**) roughness.

In the traditional high-speed and high-pressure rapid polishing process of SiC components, it was found that the surface of SiC components is prone to “white spot” defects, which can expand during subsequent processing, affecting processing accuracy and surface quality. To solve this problem, a comprehensive orthogonal comparison experiment of rapid polishing of SiC components was conducted under internal and external fluid supply conditions. Two different fluid supply methods were used in the experiment: internal and external fluid supply, with the remaining parameters including a tool diameter of Ø50 mm, the diamond abrasive particle size of 3 μm, a solution concentration of 1%, air pressure parameters of 0.15, 0.2, and 0.25 MPa, and rotational speed parameters of 500, 1000, and 1500 rpm. In the experiment, an infrared imaging camera was used to detect the temperature of the polishing area. After the experiment, the defect density of the components under different parameters was statistically analyzed, the surface defect characteristics of the components were observed under a microscope, and the surface roughness of the components was detected using a white light interferometer. The experimental results are shown in Figure 8, Figure 9 and Figure 10.

The experimental results showed that the surface defects of SiC under external fluid supply conditions were related to the process parameters, with higher rotational speeds and air pressures resulting in higher defect densities on the surface of SiC. However, under central fluid supply conditions, there were no surface defects on SiC under all process parameters. It was therefore clear that a central fluid supply can greatly suppress SiC processing defects. Figure 8 shows a comparison of surface quality under different fluid supply methods, where there are “white spot” defects in the center of the component under external fluid supply conditions but no defects on the surface of the component under internal fluid supply conditions.

Figure 9 and Figure 10 are microscopic images of the surface of SiC components processed using two different liquid supply methods, internal and external. RB-SiC is composed of silicon carbide particles and a binder (silicon), and when the surface is defect-free, the silicon carbide and binder are randomly distributed, as shown in Figure 10a. When defects appear on the surface of the silicon carbide, they exhibit a “binder erosion” characteristic, as shown in Figure 9a. Furthermore, comparing the surface roughness of the silicon carbide, it is found that the “white spots” defects are “pits”, which significantly increase the roughness (Rq 70.6 nm), as shown in Figure 9b, whereas the normal area without defects has excellent roughness results (Rq 4.45 nm), as shown in Figure 10b.

**Figure 11:**
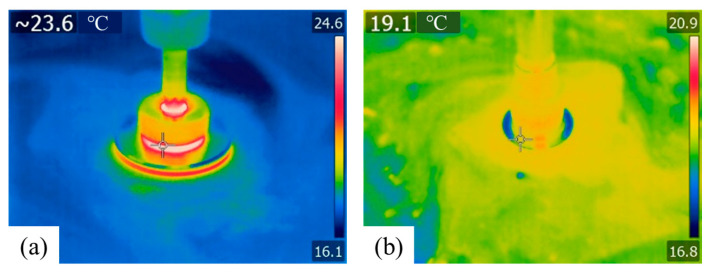
Thermal imaging of machining with different liquid supply methods. (**a**) External liquid supply; (**b**) internal liquid supply.

From Figure 11, it can be seen that under external liquid supply conditions, there is a significant amount of machining heat at the contact interface between the tool and the component (a temperature of 24.6 °C), and the temperature at the contact center may be even higher. Under internal liquid supply conditions, there is no significant temperature rise at the contact interface between the tool and the component, and the machining area is forcibly cooled by the low-temperature polishing liquid (temperature of 16.8 °C). This indicates that under high-speed and high-pressure conditions, it is difficult to polish the center region using the external liquid supply method since the heat generated during machining cannot be effectively removed. In contrast, with an internal liquid supply, the polishing liquid is updated promptly, and the polishing heat can be carried away.

In summary, the temperature rise in the machining area is the primary cause of surface defects on SiC, and it has been demonstrated that center supply polishing can significantly suppress machining heat, thereby achieving defect-free and excellent surface roughness.

### 3.3. Magnetorheological Precision Polishing and Ultra-Smooth Surfaces

**Figure 12:**
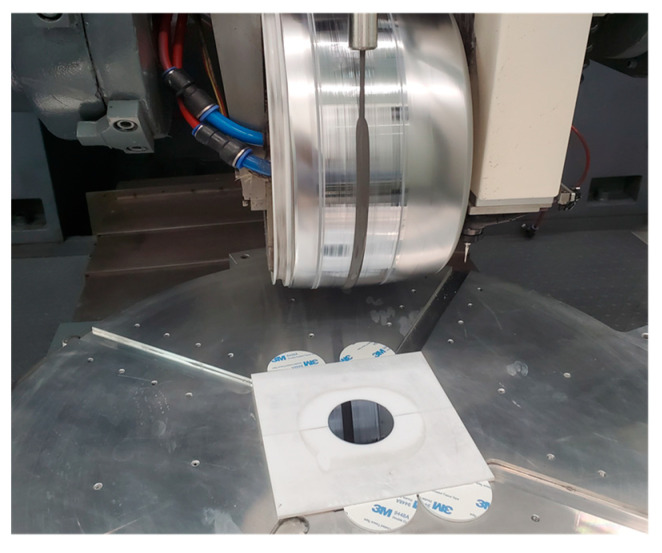
Experimental photo of magnetorheological polishing.

**Figure 13:**
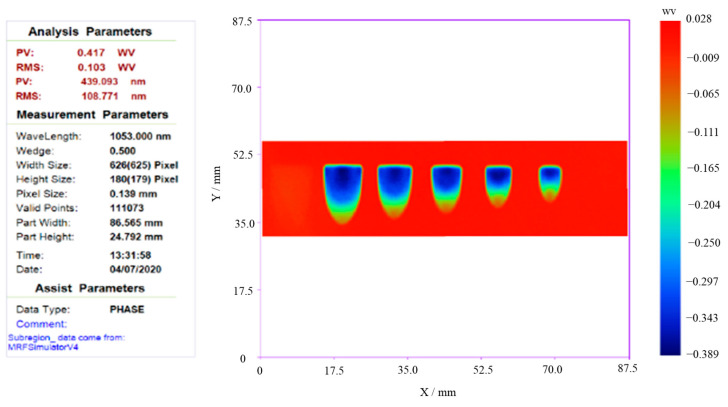
MRF spots with different scales.

The magnetorheological polishing process has high determinism, manifested in the fact that different sizes and highly stable flexible polishing spots can be obtained by selecting process parameters, making it feasible to use magnetorheological polishing to process high-precision silicon layer mirrors. In the precision polishing stage, different sizes of polishing spots can be obtained by controlling the process parameters of the magnetorheological polishing process, including immersion depth and polishing fluid flow rate, to achieve high-precision matching with different frequency band errors on the surface of the component and to accurately correct the surface shape of the component. Figure 12 and Figure 13 show real photos and results of magnetorheological polishing.

**Figure 14:**
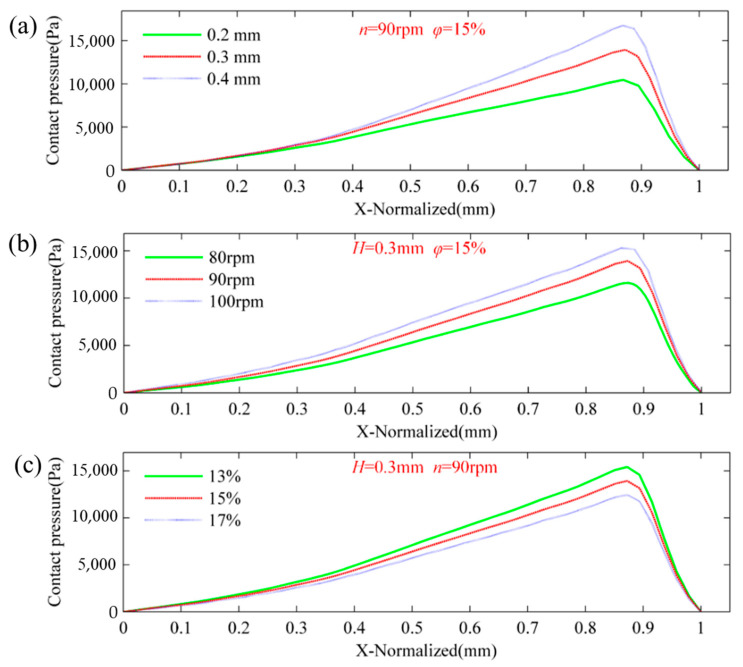
The influence curve of different process parameters on contact pressure. (**a**) Immersion depth; (**b**) rotation speed; (**c**) water content.

After completing the precision polishing, the surface shape of the component has reached the required accuracy, but the surface roughness is still insufficient. The processed mirror then enters the next process, which is ultra-smooth machining using magneto-rheological finishing (MRF). By optimizing the MRF process parameters, the processing force acting on the component can be reduced, and the surface smoothness can be improved. Aiming to achieve low-force machining, a simulation analysis was conducted to control the contact pressure between the component and the polishing spot by using a controlled variables approach for immersion depth, rotational speed, and water content, which are commonly used process parameters. The immersion depth typically ranges from 0.2 mm to 0.4 mm, and the rotational speed ranges from 80 rpm to 100 rpm. The water content that affects the viscosity of the polishing fluid is usually controlled within the range of 13–17%, and water content values of 13%, 15%, and 17% were selected for the simulation calculation within the controllable range. The influence curve of different process parameters (i.e., immersion depth, rotation speed, water content) on contact pressure are shown in Figure 14.

As shown in the above figure, to obtain a smaller contact pressure during the polishing process, it is necessary to select a lower rotational speed and immersion depth, as well as a higher water content within the controllable range. Meanwhile, changing the particle size of the polishing fluid can reduce the impact force of the fluid on the element and improve the surface quality of the element. In the process, a polishing fluid with a particle size of 50 nm is used for ultra-smooth polishing of element surfaces, which achieves rapid convergence of surface roughness while avoiding damage to the well-polished surface shape.

### 3.4. Compensation of High-Order Surface in Interferometric Measurement Using CGH

To meet the high-precision testing requirements of silicon carbide aspherical mirrors, a diffraction compensation method was used to complete the compensation design of a Ø460 mm silicon carbide aspherical element. The equation of Ø460 mm silicon carbide aspheric surface is shown in Formula 1. In this formula, *c* = −1/*R*_0_, the vertex radius *R*_0_ is 5378.6 mm and the cone coefficient *k* is 6.963. *α_i_* is the high-order aspheric coefficient; *α*_1_ = 0, *α*_2_ = 3.737 × 10^−11^, and *α*_3_ = 4.226 × 10^−17^.

(1) $$z(x,y)=\frac{c(x^{2}+y^{2})}{1+\sqrt{1-(1+k)c^{2}(x^{2}+y^{2})}}+\sum_{i=1}^{n}\alpha_{i}{\left(x^{2}+y^{2}\right)}^{i}$$

**Figure 15:**
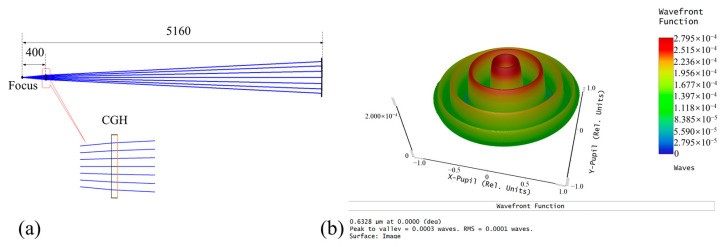
Design of the inspection optical path. (**a**) CGH compensator; (**b**) design wavefront.

Considering the characteristics of the element, such as its long focal length and lack of off-axis, a defocused carrier frequency method was used to design the computer-generated hologram (CGH) for shape measurement, and the phase parameters of the compensating element were optimized based on a circular symmetrical diffraction surface. The ideal design wavefront was obtained with a design wavefront PV of 0.0003 λ (λ = 632.8 nm), as shown in Figure 15.

**Figure 16:**
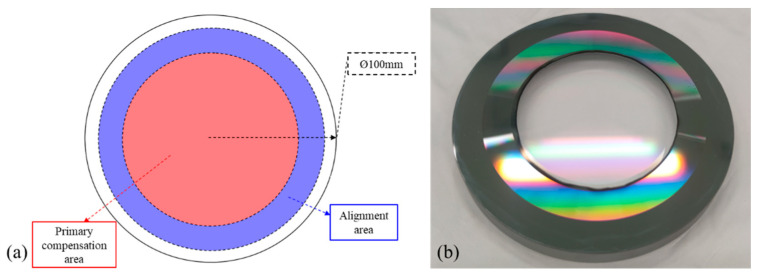
Design and fabrication of CGH compensation. (**a**) CGH design; (**b**) compensator.

The basic design and area distribution of the compensation plate are shown in Figure 16. The size of the element substrate is Ø100 mm × 10 mm, with an etching ratio of 1:1 and etching depths of 484 nm for level 1 and 152 nm for level 3. A certain ring area is reserved at the edge for mechanical clamping. The central region is the main compensation area for transmissive diffraction (radius 0–35 mm), mainly used for compensating the asphericity of the test element, with a design minimum period of ~14.18 μm. The edge annular area (radius 35–45 mm) is designed for level 3 reflection diffraction; considering the low diffraction efficiency, a high reflectivity film is deposited, and the minimum etching period is ~8.64 μm.

Microscopic inspection equipment combined with precision translation stages was used to evaluate the etching distortion error. Samples were taken at intervals along the horizontal and vertical radii and compared to the corresponding theoretical linewidth data from software simulations. The etching error of the component was then evaluated using a multi-region etch line error analysis. A binary phase-type CGH was used in the design, and the etching step depth was measured using an optical profiler. The etching step depth error was statistically analyzed by continuous sampling at 3 mm intervals. Due to the use of a first-order phase-type diffraction design, the influence of etching aspect ratio error on wavefront error can be neglected. According to the current level of etching technology, other errors such as mask encoding errors and amplitude errors caused by etching are also negligible. The overall fabrication accuracy of the diffraction compensating plate meets the design requirements.

**Figure 17:**
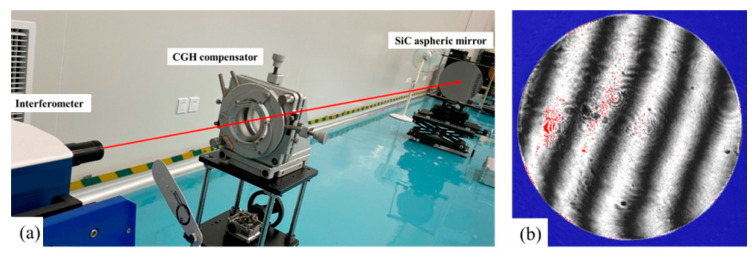
Optical path and interference fringe. (**a**) The optical path of measurement; (**b**) fringe.

Based on a computational holographic plate, a detection optical path for compensating the surface shape of a large-aperture SiC reflective mirror element was built. Using the CGH ring to assist in adjusting the region, the focus of the detection optical path and the precise alignment between the compensating element were realized, which ensured the high-precision adjustment of the detection optical path and the high-precision surface shape detection of the element, as shown in Figure 17.

## 4. Verification of the Process Chain on a Ø460 SiC Aspheric Mirror

Based on the above technical scheme and key technologies, the entire process of ultra-precision machining and inspection was carried out for a Ø460 mm carbonized silicon aspheric reflector.

### 4.1. Ultra-Precision Grinding

**Figure 18:**
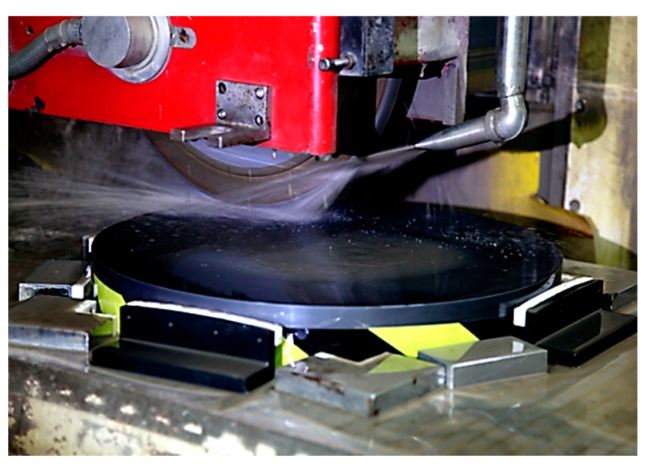
Photos of the ultra-precision grinding process.

**Figure 19:**
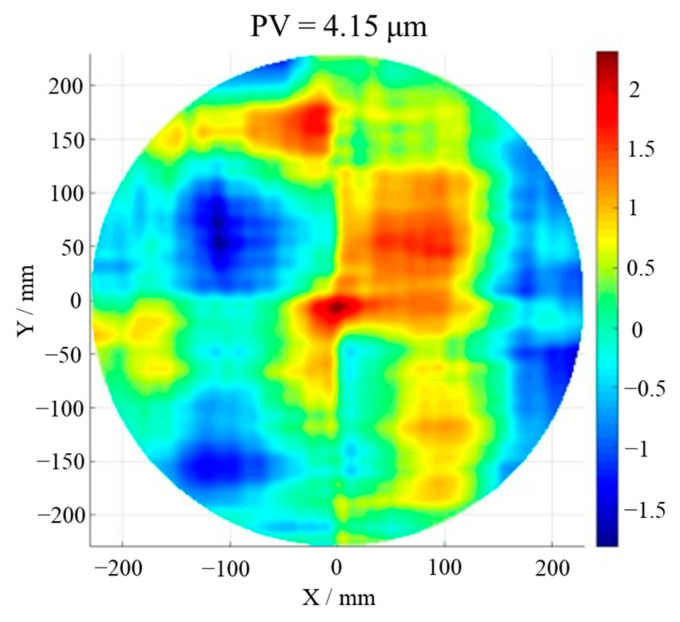
Figure of SiC aspheric mirror after grinding.

**Figure 20:**
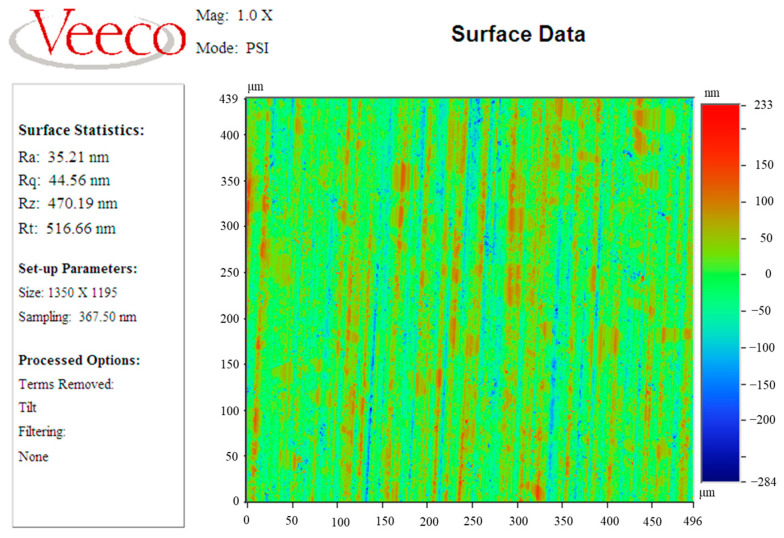
Roughness of SiC aspheric mirror after grinding.

According to the technical process, the Ø460 mm SiC aspherical mirror was the first ultra-precision part ground using an aspherical parallel grinding technique on an ultra-precision grinding machine. The diamond grinding wheel with a controlled arc section profile was used to strictly follow the aspherical shape, and the wheel was moved in a raster envelope motion along the surface of the component. The trajectory of different contact points on the outer circular contour surface of the wheel formed an aspherical envelope surface on the workpiece, completing the entire aspherical surface shaping process, as shown in Figure 18. In the shaping process, a non-contact in situ detection method was combined to control the non-contact displacement sensor to move along the surface of the component according to the aspherical shape and obtain the aspherical contour error. The three-dimensional surface shape error of the aspherical surface was determined by data postprocessing for deterministic compensation grinding. After 32 h of grinding, the final surface shape of the component converged on a PV of 4.15 μm, as shown in Figure 19. After grinding, the surface roughness of the aspherical surface was measured using a roughness tester, and the result was 44.56 nm, as shown in Figure 20.

### 4.2. Center-Supply Fluid Rapid Polishing

**Figure 21:**
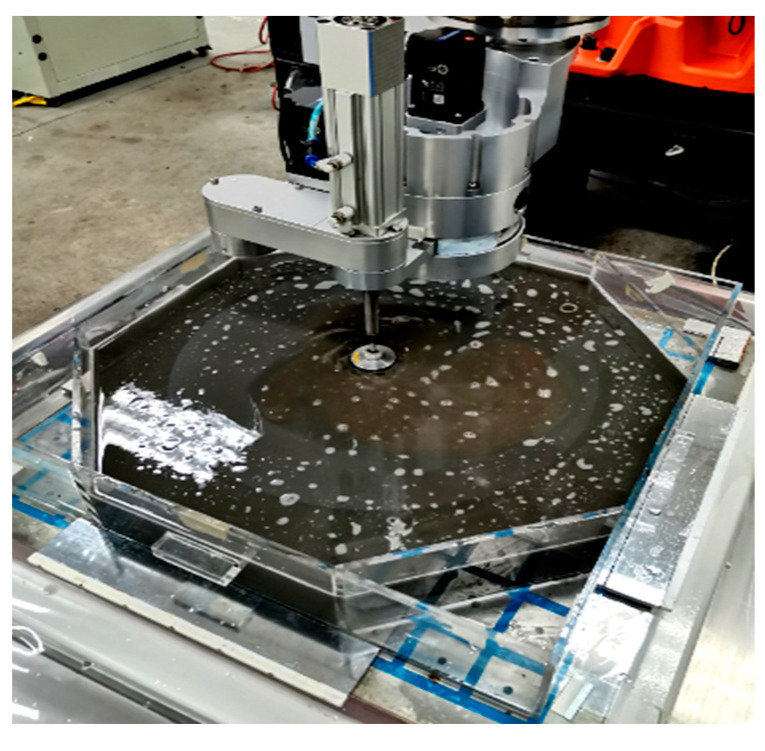
Photos of the rapid polishing process with center fluid supply.

After non-axisymmetric grinding, a combination of fixed abrasive and loose abrasive tools based on robot-centered fluid supply technology was used for shape-preserving rapid polishing of the ultra-precision ground non-axisymmetric component, removing surface defects and ripples from the grinding process, removing depths greater than 10 μm, and then quickly shaping to the precision polishing entrance accuracy. First, a 4 μm fixed abrasive polishing disk was used to quickly remove grinding ripples, followed by a 3μm loose abrasive diamond slurry and asphalt disk to further smooth residual ripples from the previous step, resulting in a surface that can be entered into interferometric testing. Next, a 3 μm loose abrasive diamond slurry and polyurethane disk were used to shape the surface. The process of robot-centered fluid supply for small tool rapid polishing is shown in Figure 21.

**Figure 22:**
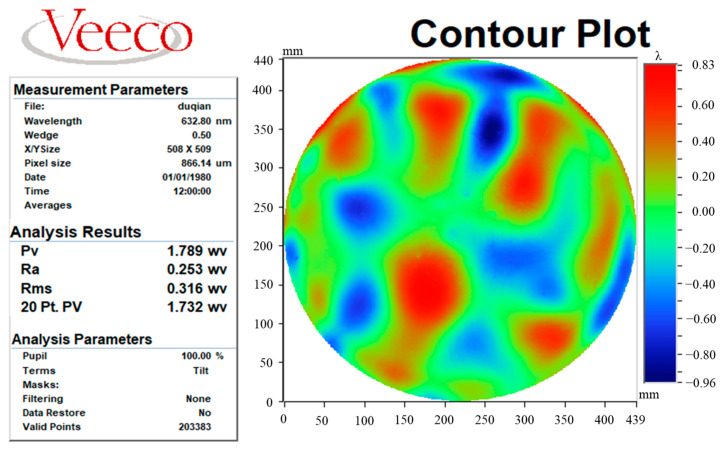
Surface form error of the SiC aspheric mirror after CCOS.

**Figure 23:**
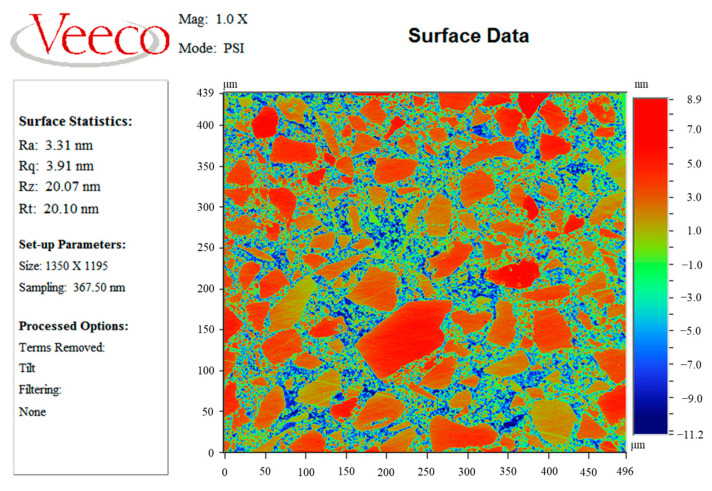
Roughness of the SiC aspheric mirror after CCOS.

During the polishing process, a high-order surface mirror CGH compensation interferometric detection optical path (as shown in Figure 17) was constructed using a spherical interferometer and a CGH compensator for aspherical surface shape detection, which guided subsequent surface shape correction. After approximately 160 h of rapid polishing with a center supply liquid tool, the surface shape error was significantly improved, with a PV of 1.78 λ and an Rq surface roughness of 3.91 nm for the aspherical mirror, as shown in Figure 22 and Figure 23.

### 4.3. Deterministic and Ultra-Smooth Polishing by MRF

**Figure 24:**
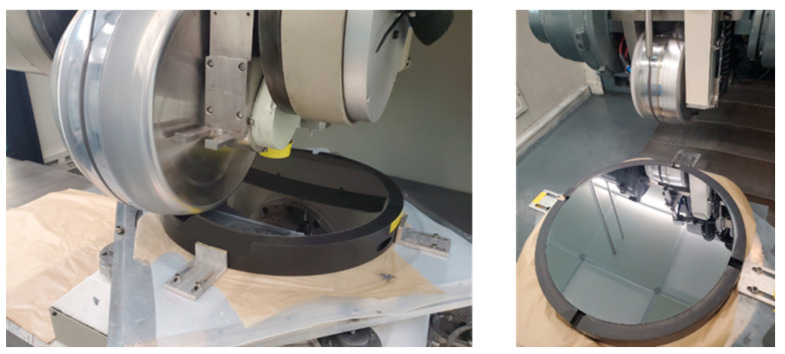
Photographs of the MRF process.

**Figure 25:**
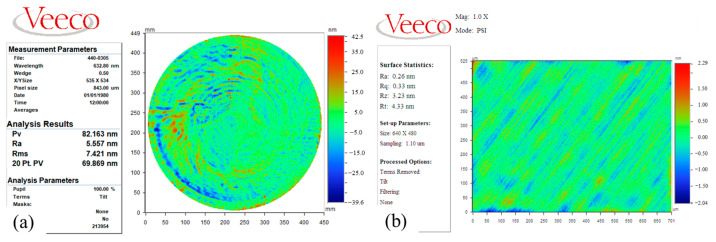
Result of the SiC aspheric mirror after MRF. (**a**) Wavefront; (**b**) surface roughness.

After the rapid polishing, the surface of the element was modified by silicon plating with a thickness of about 15 μm, and the surface shape was kept unchanged. Then, the magneto-rheological polishing technology was used for aspherical element shape correction and ultra-smooth polishing, as shown in Figure 24. Based on the basic research of magneto-rheological processing of silicon materials, the magneto-rheological removal function control and ultra-smooth surface polishing technologies were adopted. After three cycles of about 24 h of magnetorheological finishing, the high-precision and high-quality processing of the silicon-plated surface of the Φ460 mm aspherical reflector was achieved. The surface shape was PV 82.16 nm, RMS 7.42 nm, and roughness Rq 0.33 nm, as shown in Figure 25. The experimental results indicate that magneto-rheological polishing not only has a strong surface shape correction ability but also can achieve ultra-smooth polishing of silicon surfaces, thus realizing high-precision and ultra-smooth precision polishing of SiC aspheric mirrors.

## 5. Conclusions

This paper proposes an efficient manufacturing process chain for silicon carbide aspheric mirrors that combines ultra-precision grinding, rapid polishing, central fluid supply, and magnetorheological finishing (MRF). This process chain was validated on a Ø460 silicon carbide aspherical mirror with an initial surface shape error for PV of 4.15 μm and a roughness of Rq of 44.56 nm. The surface error of RMS 7.42 nm and roughness Rq of 0.33 nm were successfully obtained, with a total manufacturing cycle of only 216 h, which indicates the feasibility of the proposed process chain for the high-precision and high-efficiency manufacturing of the large-aperture SiC aspheric mirrors. The manufacturing cycle of the proposed process chain would be an effective method for the mass production of large-aperture SiC aspheric mirrors used in space optical remote sensing detection systems, high-resolution ground observation systems, space telescopes, etc. In addition, to further improve the fabrication efficiency of SiC mirrors, manufacturing techniques such as laser-assistance [28,29] and vibration-assistance [30,31] may be integrated into current process chains in the future.

## Data Availability

Not applicable.
